# Relative and attributable risks of neurological and perinatal adverse outcomes among children with and without prenatal Zika virus exposure in Northeast Brazil: A prospective cohort study (2015–2018)

**DOI:** 10.1371/journal.pntd.0013344

**Published:** 2025-08-08

**Authors:** Juliana Menezes Soares de Souza Azevedo Fontes, Demócrito de Barros Miranda-Filho, Ulisses Ramos Montarroyos, Thalia Velho Barreto de Araújo, Celina Maria Turchi Martelli, Laura Cunha Rodrigues, Elizabeth Brickley, Maria de Fátima Pessoa Militão de Albuquerque, Wayner Vieira Souza, Liana Maria Vieira de Oliveira Ventura, Camila Vieira de Oliveira Ventura, Adriana Lima Gois, Mariana de Carvalho Leal Gouveia, Danielle Maria da Silva Oliveira, Sophie Helena Eickmann, Maria Durce Costa Gomes Carvalho, Paula Fabiana Sobral da Silva, Maria Ângela Wanderley Rocha, Regina Coeli Ferreira Ramos, Sinval Pinto Brandão-Filho, Marli Tenorio Cordeiro, Luciana Caroline Albuquerque Bezerra, George Santiago Dimech, Sandra Valongueiro Alves, Pedro Pires Ferreira Neto, Priscila Mayrelle da Silva Castanha, Rafael Dhalia, Ernesto Torres de Azevedo Marques, Gabriela Renata Neves Fulco, Maria Valquíria de Medeiros Silva, Ricardo Arraes de Alencar Ximenes, and on behalf of the Microcephaly Epidemic Research Group (MERG)

**Affiliations:** 1 Programa de Pós-graduação em Ciências da Saúde, Universidade de Pernambuco, Recife, Pernambuco, Brazil; 2 Faculdade de Ciências Médicas, Universidade de Pernambuco, Recife, Pernambuco, Brazil; 3 Instituto de Ciências Biológicas, Universidade de Pernambuco, Recife, Pernambuco, Brazil; 4 Departamento de Medicina Social, Universidade Federal de Pernambuco, Recife, Pernambuco, Brazil; 5 Departamento de Saúde Coletiva, Instituto Aggeu Magalhães, Recife, Pernambuco, Brazil; 6 Department of Infectious Disease Epidemiology, London School of Hygiene & Tropical Medicine, London, United Kingdom; 7 Departamento de Oftalmologia, Fundação Altino Ventura, Recife, Pernambuco, Brasil; 8 Departamento de Cirurgia, Universidade Federal de Pernambuco, Recife, Pernambuco, Brazil; 9 UTI Pediátrica, Hospital Universitário Oswaldo Cruz, Recife, Pernambuco, Brazil; 10 Departamento Materno-Infantil, Universidade Federal de Pernambuco, Recife, Pernambuco, Brazil; 11 Departamento de Neurologia, Hospital Universitário Oswaldo Cruz, Recife, Pernambuco, Brazil; 12 Departamento de Infectologia Pediátrica, Hospital Universitário Oswaldo Cruz, Recife, Pernambuco, Brazil; 13 Departamento de Imunologia, Instituto Aggeu Magalhães, Recife, Pernambuco, Brazil; 14 Laboratório de Virologia e Terapia Experimental, Instituto Aggeu Magalhães, Recife, Pernambuco, Brazil; 15 Secretaria de Saúde do Recife, Recife, Pernambuco, Brazil; 16 Secretaria Executiva da Vigilância em Saúde, Secretaria de Saúde de Pernambuco, Recife, Pernambuco, Brazil; 17 Departamento Materno-Infantil, Universidade de Pernambuco, Recife, Pernambuco, Brazil; 18 Departamento de Medicina Tropical, Universidade Federal de Pernambuco, Recife, Pernambuco, Brazil; The University of the West Indies, JAMAICA

## Abstract

**Background:**

Although there has been substantial progress in the characterization of Congenital Zika Syndrome, the lack of a control group in the majority of published studies on Zika virus (ZIKV) infections during pregnancy limits our understanding of, first, the magnitude by which prenatal ZIKV exposure may increase risks of adverse outcomes for offspring and, second, the fraction of abnormalities that are attributable to this exposure.

**Methods:**

To overcome this limitation, this study harmonized and integrated data collected prospectively in Recife, Pernambuco, Brazil, from offspring of ZIKV-exposed women in the Microcephaly Epidemic Research Group (MERG) Pregnant Women Cohort and from offspring of ZIKV-unexposed women in the Zika in Infants and Pregnancy (ZIP) Study. We compared the data to estimate the relative risk (RR) and attributable risk percent (AR%) of: (i) adverse birth outcomes including low birth weight (LBW), prematurity and small for gestational age (SGA) and (ii) developmental abnormalities including microcephaly and neurological, ophthalmological, audiological, and neuroimaging alterations.

**Findings:**

We observed similar odds of adverse birth outcomes and ophthalmological deficits in ZIKV-exposed and unexposed children. However, as compared to ZIKV-unexposed children, ZIKV-exposed children presented with markedly increased risks of microcephaly (RR, 95%-CI: 3.61, 1.70 to 7.63 AR 72%), neurological abnormalities (RR, 95%-CI: 5.64, 3.04 to 10.47.79AR 82%), audiological screening failures (RR, 95%-CI: 9.20, 2.59 to 32.69 AR 89%), and neuroimaging abnormalities (RR, 95%-CI: 22.06, 2.90 to 167.5; AR 95%). The risk of having concurrent abnormalities was lower than the risk of having just one abnormality. Our results provide new insights into the relative and attributable risks related to prenatal ZIKV exposure and demonstrate that, overall, the risks of congenital abnormalities are elevated among children exposed to ZIKV during pregnancy compared to their ZIKV-unexposed peers.

## Author summary

Since the start of microcephaly epidemic in 2015, knowledge has accumulated regarding the spectrum of clinical manifestations resulting from congenital Zika virus infections and the frequency with which they occur. However, it remains unclear how much higher the risk of abnormalities is among children born to women who were infected with Zika virus during pregnancy when compared with children born to women who were not infected during pregnancy. We compared these two groups and found prenatal ZIKV exposure was associated with markedly increased risks of microcephaly (RR, 95%-CI: 3.61, 1.70 to 7.63, AR 72%), neurological abnormalities (RR, 95%-CI: 5.64, 3.04 to 10.47; AR 82%),), audiological deficits (RR, 95%-CI: 9.20, 2.59 to 32.69; AR 89%), and neuroimaging alterations (RR, 95%-CI: 22.06, 2.90 to 167.5; AR 95%). We found that at least 41% of cases of microcephaly, 67% of cases with neurological alterations and 65% of the cases with CNS imaging alterations among children born to exposed mothers may be attributed to the prenatal ZIKV infection. Overall, these findings underscore the importance of continuing the follow-up of these children to evaluate the long-term consequences of ZIKV infection during pregnancy and reinforce the need to rapidly develop a safe and effective vaccine to prevent congenital ZIKV infections.

## Introduction

Confirming the causal link between Zika virus (ZIKV) infection during pregnancy and microcephaly was a crucial milestone in the scientific response to the 2016 Public Health Emergency of International Concern [1]. Early observational studies also recognized that congenital ZIKV infections could be associated with a range of malformations and developmental deficits, collectively known as Congenital Zika Syndrome (CZS) [2]. Microcephaly and other neurological abnormalities may be explained by ZIKV’s neurotropism [3]. Although knowledge on ZIKV has rapidly advanced since 2015, comprehensive observational epidemiological research investigating adverse health outcomes associated with prenatal ZIKV infection have continued to encounter challenges, owing primarily to the small sample sizes of individual cohorts [4,5] and the lack of robust comparator populations [6]. To overcome the sample size limitation and facilitate a better understanding of heterogeneity between study populations, research consortia including the Zika Brazilian Cohorts (ZBC) Consortium [7] and the World Health Organization-led ZIKV Individual Participant Data Consortium [8] were formed. However, conducting meta-analyses is not without challenges due to the high level of cross-study and within-study heterogeneity in both maternal ZIKV exposure and outcome ascertainment [9].

Few studies have compared the risk of adverse outcomes between children born to mothers who were exposed and those who were unexposed to ZIKV during pregnancy. In a cohort study that recruited pregnant women with rash in Rio de Janeiro, Brazil, Brasil and colleagues (2016) reported that, relative to the ZIKV-negative group, the ZIKV-positive group experienced similar rates of fetal death (7.2% in ZIKV-positive vs. 6.6% in ZIKV-negative) but their liveborn offspring experienced elevated risks of overall adverse outcomes (41.9% vs. 5.3%), being small for gestational age (SGA, 8.6% vs 5.3%), and having microcephaly (3.4% vs. 0%) [4]. In a cohort study based in a high-risk pregnancy clinic in São Paulo, Brazil, Sanchez Clemente and colleagues (2020) observed, that relative to children born to ZIKV-negative women, children born to ZIKV-positive women had similar risks of low birth weight (<2500g, 9.1% in ZIKV-positive vs. 11.1% in ZIKV-negative) and of being small for gestational age (9.1% vs. 9.9%) but non-significantly higher risks of microcephaly (4.5% vs. 1.9%, RR 2.3, 95%-CI 0.5 to 10.3) [5]. In a cross-sectional study in Guadeloupe, Funk and colleagues (2021) compared offspring of ZIKV-positive women previously recruited as part of a prospective cohort study to those of ZIKV-negative women recruited at delivery and reported no statistically significant differences in the overall risks of “any neurological or ocular abnormalities” between the groups (6.6% in ZIKV-positive vs. 8.6% in ZIKV-negative) [6]. Relative risks have also been explored in a limited number of registry-based studies. Using surveillance data on travelers from the International Zika in Pregnancy registry, Vouga and colleagues (2021) reported higher frequencies of severe adverse pregnancy outcomes (i.e., severely affected fetuses/newborns and/or fetal loss) among travelers with evidence of recent maternal ZIKV infection as compared to uninfected travelers (8.3% in ZIKV-positive vs. 3.7% in ZIKV-negative) [9]. Overall, the published studies estimating the relative risks associated with exposure to ZIKV during pregnancy present differences in results, and the findings need to be interpreted with caution due to the small sample sizes (and the consequent lack of statistical significance in several of the comparisons) and/or methodological differences (e.g., differences in study populations and follow-up).

To advance understanding of the relative risks of adverse outcomes among children with and without prenatal ZIKV exposure, this study harmonized and integrated data collected prospectively in Recife, Pernambuco, Brazil, from offspring of ZIKV-exposed women in the Microcephaly Epidemic Research Group (MERG) Pregnant Women Cohort and from offspring of ZIKV-unexposed women in the Zika in Infants and Pregnancy (ZIP) Study. We compared the data to estimate the odds ratio (OR) and attributable risk percent (AR%) of: (i) adverse birth outcomes including LBW, prematurity and SGA and (ii) developmental abnormalities including microcephaly and neurological, ophthalmological, audiological, and neuroimaging alterations. This study advances on previous studies because of the larger sample size of unexposed pregnant women as well as the greater accuracy in identifying ZIKV-unexposed pregnant women, achieved through repeated ZIKV testing during pregnancy.

## Materials and methods

### Ethics statement

The MERG Pregnant Women’s Cohort study was approved by the Research Ethics Committee of the Aggeu Magalhães/ Fiocruz Research Center (CAAE: 53240816.4.0000.5190). The Zika in Infants and Pregnancy (ZIP) Cohort study was approved by the Research Ethics Committee of the Aggeu Magalhães/ Fiocruz Research Center (CAAE: 56673616.3.2001.5190). Both studies followed the ethical procedures recommended by Brazilian Resolution MS/CNS 466/2012. Free and informed written consent was requested for all mothers/legal guardians of participating children. Pregnant women under the age of 18 who were included in the study signed the assent form and a legal guardian signed the consent form.

### Study design and data collection

In this prospective cohort study, ZIKV-exposed pregnant women with rash were recruited from December 2015 to June 2017 in the MERG Pregnant Women’s Cohort and ZIKV-unexposed pregnant women were recruited from October 2016 to September 2018 in the ZIP Cohort. The two cohorts compared in this study were recruited from the same geographic region (i.e., residing up to 120km from the Metropolitan Region of Recife) and were followed up by the same research group (MERG). For both groups, the exposure was ZIKV infection during pregnancy and the outcomes were adverse outcomes for the pregnancy and offspring. For the children in the MERG Cohort, the outcome data that were analyzed by the research team were collected either at birth or at first assessment. For children in the ZIP Cohort, the outcome data that were analyzed were collected either at birth or at an assessment at an age closest to that at which the MERG children were assessed.

The MERG Cohort was formed of pregnant women, at any age, who were notified with rash by the health units to the State of Pernambuco Health Surveillance Strategic Information Center - Cievs/ PE. Notification of pregnant women with rash was made compulsory by the Pernambuco Health Department since December 2015. Blood samples were collected within five days of rash and at least 14 days after notification, the MERG fieldworkers recruited these women into this study, collected a second blood sample and administered a standardized questionnaire and, in cases of livebirths, a third blood sample was collected after delivery. [10] For the current analysis, only those who presented with laboratory evidence of ZIKV infection during pregnancy and were considered ZIKV-exposed were included. The ZIP cohort consisted of women recruited in the first and second trimester (i.e., up to 17 weeks and 6 days of gestation) or presenting with acute Zika-like symptoms and laboratory confirmed ZIKV infection by serology or RT-PCR at any gestational age. Study recruitment was done sequentially in public prenatal clinics of the Sistema Único de Saúde (Unified Health System) in the city of Recife. A total of 33 prenatal clinics located in different parts of the city were selected. For this study, only women from the ZIP Cohort who repeatedly tested negative for ZIKV during pregnancy and were considered ZIKV-unexposed were included. Pregnant women aged less than 15 years were excluded.

### ZIKV exposure during pregnancy

ZIKV exposure during pregnancy was based on laboratory evidence. In the MERG Cohort maternal sera were tested for the detection of ZIKV RNA by one-step qRT-PCR, diagnostic ZIKV-specific IgM antibodies, ZIKV-specific IgG3 anti-non-structural protein 1 (NS1) and PRNT, and we categorized maternal ZIKV exposure according to the degree of evidence of infection, as previously described. (see Ximenes et al. 2019 [10] and Ximenes et al. 2021 [11]) for detailed description). Pregnant women were first classified as positive or suspected for ZIKV infection. The positive group was divided into three subcategories according to the level of diagnostic evidence (i.e., robust, moderate and limited evidence). The suspected ZIKV infection group included two subcategories (i.e., limited evidence of flavivirus exposure and inconclusive results). Analyses was performed first by grouping positive and suspected cases together and, then as a sensitivity analysis, restricting to the ZIKV-positive cases alone [10]. In the ZIP Cohort maternal participants were tested by rRt-PCR, Anti-ZIKV IgM antibodies. Blood samples for pregnant women were collected on a monthly basis and tested for Anti-ZIKV IgM antibodies. If a woman tested positive by serology, the Rt-PCR was performed on the monthly blood sample that was collected on or between the dates of two blood samples and used in the serologic test. As previously described, the ZIKV-unexposed group was defined in the ZIP study as individuals who repeatedly tested negative for ZIKV during pregnancy. Pregnant women in the ZIP Cohort were tested a median of eight times during pregnancy. For Anti-ZIKV immunoglobulin (Ig)M antibodies, we used a serological assay with Emergency Use Authorization (EUA) approval from the United States Food and Drug Administration (FDA); in the event of a routine monthly visit blood sample testing positive, a follow-up EUA-approved ZIKV molecular assay (quantitative reverse transcription polymerase chain reaction (qRT-PCR)) was performed for the detection of ZIKV RNA [12].

### Outcomes

Although the cohorts were followed up by the same research group, there were some differences in the questionnaires and assessments applied. Therefore, for the current investigation, it was necessary to harmonize the data and standardize the variables prior to the joint analyses. We first selected the variables that were assessed in both cohorts and then we categorized these variables in the same way. No matching was performed for the analysis.

The adverse pregnancy outcomes included: *low birth weight* (defined as both weight < 2500g and ≤2 standard deviations (SD) below the mean), *prematurity* (defined as <37 weeks gestational age), and *SGA* (defined as birth weight <10^th^ percentile for gestational age). *Microcephaly,* was defined as a head circumference measuring ≥2 SDs below the mean for age and sex, based on the World Health Organization growth curves for infants born at term and on the INTERGROWTH-21st curves for infants born at <37 weeks of gestational age [11]. We performed a sensitivity analysis for microcephaly considering alternative cutoff points of 2, 2.5, and 3 SDs below the mean. *Neurological abnormalities* were defined to include: irritability, altered tonus, dysphagia and seizures. *Ophthalmological abnormalities* were defined to include: alterations in the fundus, optic nerve (hypoplasia, pallor or papillary excavation) and/or retina (pigment dispersion, chorioretinal atrophy, vascular alteration) identified in the RetCam (Natus Medical Incorporated, Middleton, WI, USA) ophthalmic imaging examination. *Failure in the audiological screening test* was based on the results of the brainstem evoked response audiometry (BERA); as it was a screening test the results were categorized into failure or non-failure, at the first evaluation. *Central nervous system (CNS) imaging alterations* were defined using transfontanellar ultrasound based on the presence of calcifications and ventriculomegaly.

In addition to evaluating the risk of each isolated abnormality (i.e., microcephaly, neurological abnormalities, ophthalmological abnormalities, and CNS imaging alterations), we also evaluated the risk of having at least one these abnormalities as well as children’s risks of having concurrent alterations in three combinations: (i) microcephaly, neurological abnormalities, and CNS imaging alterations; (ii) neurological abnormalities and CNS imaging alterations; and (iii) neurological and ophthalmological abnormalities. Hearing abnormalities were not included as the outcomes in this study were based on a screening rather than a diagnostic assessment.

### Sociodemographic characteristics and potential cofounders

The following sociodemographic and clinical characteristics were compared between the ZIKV-exposed and unexposed women: maternal age, race/ethnicity, years of education, number of previous pregnancies, having children with malformations from previous pregnancies, smoking, recreational drug use and delivery mode. Those variables that presented an association with the exposure and for which there was a plausible association with the outcomes were considered potential cofounders and were adjusted for in the analysis. In both cohorts, a subsample of pregnant women was tested for Rubella, Cytomegalovirus and Toxoplasmosis. In the MERG Cohort, a subsample of pregnant women was also tested for Parvovirus B19.

### Statistical methods

In the data analysis, sociodemographic characteristics and pregnancy outcomes of ZIKV-exposed and unexposed mothers were compared using Pearson’s Chi-squared tests for categorical variables and Student’s t or Mann-Whitney U tests for continuous variables. In the bivariate analysis, for each of the adverse outcomes studied, the relative risk and the attributable risk percent (AR%, the percentage of the cases among exposed that can be attributed to the exposure) were estimated with a 95% confidence interval. To deal with zero cells for some variables, the OR was calculated using a median unbiased estimator for binary data in an unconditional logistic regression model [13,14]. As the populations differed by some baseline characteristics, we estimated the adjusted ORs. In the multivariate analysis, we preferred to use the OR as the measure of association as analyses based on relative risk when examining more than one exposure variable, can cause computational problems and are difficult to interpret, The ORs pose no computational problems for regression modelling and adjusting for confounders. As a sensitivity analysis, we re-calculated the ORs restricting the ZIKV-exposed group to ZIKV-positive participants with robust, moderate, or limited laboratory evidence of maternal infection. The significance level adopted was 5% (p < 0.05), and data were analyzed using Stata version 14 (StataCorp LLC, College Station, TX, USA).

## Results

**Fig 1:**
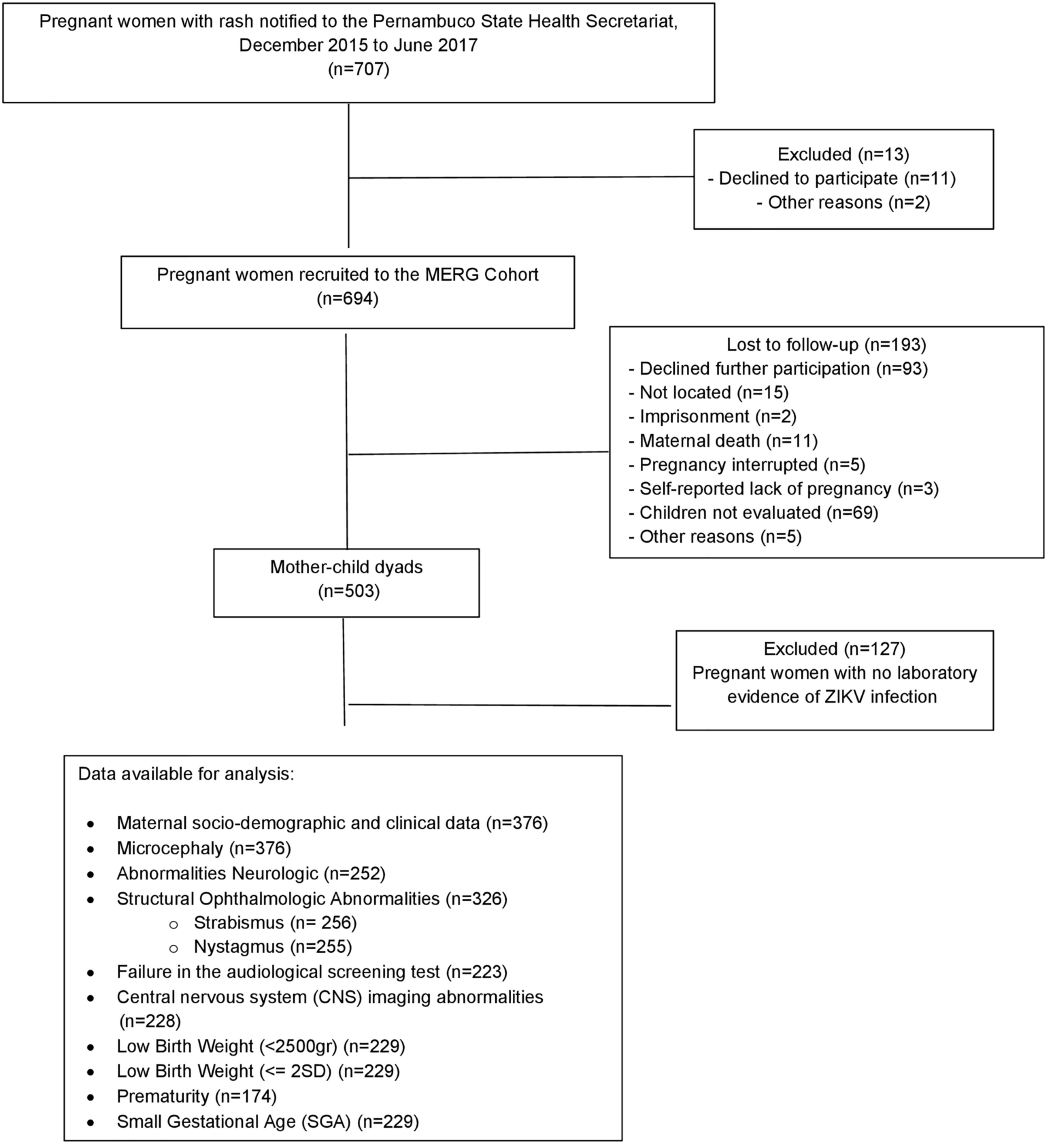
Flow diagram of the MERG Pregnancy Cohort in Pernambuco, Brazil (2015–2017). Adapted from Ximenes RAdA, Miranda-Filho DdB, Montarroyos UR, Martelli CMT, Araújo TVBd, Brickley E, et al. (2021) Zika-related adverse outcomes in a cohort of pregnant women with rash in Pernambuco, Brazil. PLoS Negl Trop Dis 15(3): e0009216. https://doi.org/10.1371/journal.pntd.00092167.

**Fig 2:**
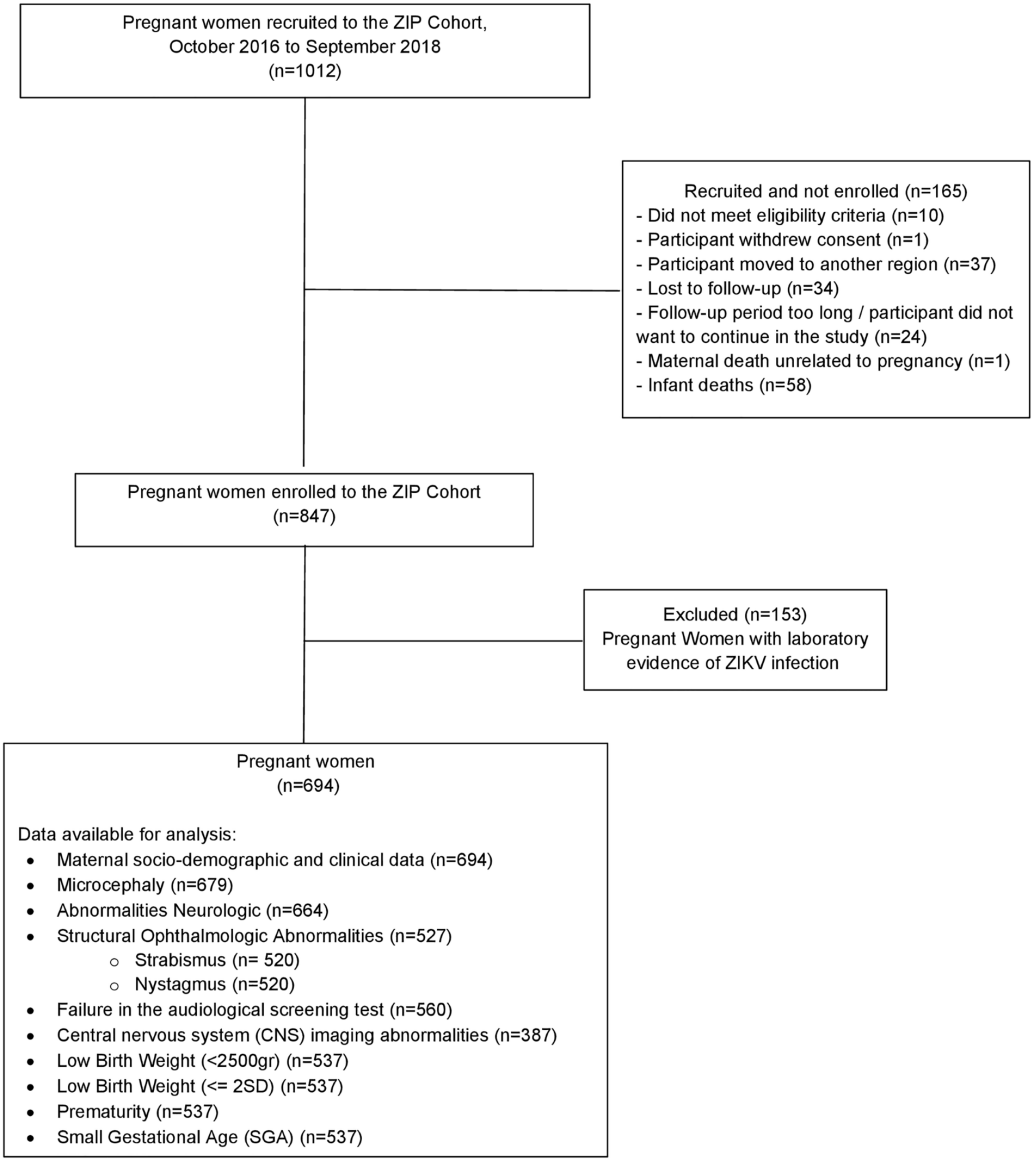
Flow diagram of the ZIP Cohort in Pernambuco, Brazil (2016–2018).

The study included 376 ZIKV-exposed and 694 ZIKV-unexposed pregnant women and their offspring (Figs 1 and 2). The characteristics of ZIKV-exposed and ZIKV-unexposed pregnant women are shown in Table 1. There were no differences in maternal age, race/ethnicity, or the frequency of malformations in previous pregnancies between the groups. Relative to the ZIKV-unexposed women, ZIKV-exposed women had lower levels of education, were more likely to smoke, less likely to use recreational drugs, and more likely to having had a previous pregnancy. Children’s age at assessment (i.e., selected as a confounder *a priori*), maternal education, smoking, recreational drug use, and previous pregnancies were considered potential cofounders and adjusted for in subsequent analyses.

**Table 1:** Characteristics of women in the MERG Pregnancy Cohort (ZIKV-exposed) and in the ZIP Cohort (ZIKV-unexposed), in Pernambuco, Brazil (2015–2020).

Variables		EXPOSED		UNEXPOSED		P value*
	Total	Median	IQR	Median	IQR	
**Age, years, Median (IQR)**		26	21-31	24	20-29	0.102
**ZIKV diagnosis in pregnancy**	**Total**	**n**	**(%)**	**N**	**(%)**	--
** ** *ZIKV - positive*	376	376	**100**	0	0	
** ** *Robust evidence, PCR and/or serology*	162	162	**43.09**	0	0	
** ** *Moderate evidence*	28	28	**7.45**	0	0	
** ** *Limited evidence*	88	88	**23.40**	0	0	
Unspecified flavivírus-positive	78	78	**20.74**	0	0	
Inconclusive	20	20	**5.32**	0	0	
Race/ethnicity**						0.323
** **“Branco” (i.e., white)	243	79	**21.01**	164	**23.67**	
** **“Não Branco” (i.e., not white)	826	297	**78.99**	529	**76.33**	
**Years of education**						<0.001
** **0-9	303	146	**38.83**	157	**22.62**	
** **10-12	621	188	**50**	433	**62.39**	
** ** + 13	146	42	**11.17**	104	**14.99**	
**Previous pregnancy**						<0.001
** **Yes	623	247	**65.69**	376	**54.18**	
** **No	447	129	**34.31**	318	**45.82**	
**Children with malformations from previous pregnancies**						0.311
** **Yes	17	8	**2.13**	9	**1.31**	
** **No	1045	368	**97.87**	677	**98.69**	
**Smoking**						0.006
** **Yes	57	29	**7.71**	26	**3.80**	
** **No	876	347	**92.29**	659	**96.20**	
**Recreational drug use**						0.037
** **Yes	29	5	**1.33**	24	**3.51**	
** **No	1031	371	**98.67**	660	**96.49**	
**Delivery mode**						0,043
** **Cesarean	489	227	**46.0**	262	**39.1**	
** **Vaginal	670	266	**53.8**	404	**60.3**	
** **Forceps	5	1	**0.2**	4	**0.60**	

*P values for categorical variables are from Chi-squared tests, and p values for continuous variables are from Mann-Whitney U tests.

**The subcategories of race/ethnicity used in the MERG cohort and in the ZIP cohort did not match, so were re-categorized as white and not white.

Table 2 provides information on the risk of microcephaly estimated using three different z-score cutoffs and the different levels of evidence of exposure (positive+suspected and positive). Considering microcephaly at birth or at first assessment and the cutoff points of -2SD, -2.5SD, and -3SD, we observed that, relative to children of ZIKV-exposed mothers, children of ZIKV-exposed mothers had more than two-times higher odds of microcephaly and that the ORs were increased using the -2.5SD and -3SD cut-offs. We estimated the AR% for microcephaly to be approximately 70%, indicating that 70% of the cases of microcephaly among the ZIKV-exposed offspring were attributable to the ZIKV infection during pregnancy. When considering microcephaly at any time during follow-up (i.e., up to approximately 18 months of age), the relative risk of microcephaly were approximately four-times higher in the ZIKV-exposed versus unexposed children. Overall, the unadjusted and adjusted ORs were similar. Of the 10 children with microcephaly born to mothers in the unexposed group, 1 presented with Edwards Syndrome (trisomy 18) and another with another chromosomal syndrome. The other eight children presented with no other clinical manifestations consistent with CZS.

**Table 2:** Relative risk, attributable risk percent and adjusted odds ratio and respectives 95% confidence intervals for microcephaly at different degrees of severity, related to Zika virus exposure during pregnancy in the MERG Pregnancy (ZIKV-exposed) cohort and the ZIP cohort (ZIKV-unexposed), in Pernambuco, Brazil (2015–2020).

Microcephaly	TOTAL	Case (N/ %)	RR (95% - CI)	P-value^#^	AR%	95% CI	OR_adjusted*_ (95% - CI)	P-valueª
**-2SD**								
*Unexposed*	679	7 **(1.03**)	1	–	–	–	–	–
*Positive + Suspected*	376	11 (**2.93**)	2.83 (1.1 -7.25)	0.022	64%	9.8% - 86%	3.27_a_ (1.21 – 8.83)	0.019
*Positive*	278	7 (**2.52**)	2.44 (0.86 – 6.89)	0.081	59%	-15% - 85%	2.59_a_ (0.83 – 8.05)	0.099
**-2.5SD**								
*Unexposed*	679	4 (**0.59**)	1	–	–	–	–	–
*Positive + Suspected*	376	8 (**2.13**)	3.61 (1.09 – 11.91)	0.024	72%	8.6% - 91%	3.76_b_ (1.08 – 13.12)	0.037
*Positive*	278	6 (**2.16**)	2.40 (0.84 – 6.88)	0.090	58%	-18% - 85%	3.50_b_ (0.96 – 12.8)	0.057
**-3SD**								
*Unexposed*	679	3 (**0.44**)	1	–	–	–	–	–
*Positive + Suspected*	376	6 (**1.60**)	3.61 (0.9 – 14.35)	0.051	72%	8.6% - 91%	4.25_b_ (1.01 – 17.8)	0.047
*Positive*	278	4 (**1.44**)	3.25 (0.73 -14.45)	0.100	69%	-36% - 93%	3.87_b_ (0.83 – 18.04)	0.085
-2SD**								
*Unexposed*	679	10 **(1.47**)			–			
*Positive + Suspected*	376	20 (**5.32**)	3.61 (1.70 – 7.63)	0.000	72%	41% - 86%	4.43_c_ (1.96 – 10.01)	<0.001
*Positive*	278	15 (**5.40**)	3.66 (1.66 – 8.05)	0.000	72%	39% - 87%	4.40_c_ (1.86 – 10.4)	0.001

^#^Pearson chi-squared test.

Positive + Suspected: Robust, moderate, limited evidence of infection + unspecified flavivirus + inconclusive evidence

Positive: - Robust, moderate, limited evidence of infection

^a^adjusted for age, years of education, recreational drug use

^b^adjusted for age and years of education

^c^adjusted for years of education, smoking and recreational drug use

**analysis considering microcephaly at any time during the follow-up

Table 3 presents the RRs, AR%s and adjusted OR of other adverse outcomes. For the presence of at least one neurological alteration related to exposure to ZIKV, the RR was 5.64 (95%-CI 3.04 to 10.47), the AR% was 82% and the OR_adj_ was 6.95 (95%-CI 3.5 to 13.79).The analysis of each neurological alteration separately (dysphagia, irritability, seizure and tonus alterations) in shown in S1 Table.). Among those that presenting a statistically significant association with the exposure (dysphagia, irritability and tonus alterations), the relative risk ranged from 5.91 for irritability to 8.91 for dysphagia. And AR% ranged from 83% for irritability to 88% for dysphagia..

**Table 3:** Relative risk, attributable risk percent and adjusted odds ratio and respectives 95% confidence intervals for adverse outcomes related to Zika virus exposure during pregnancy in the MERG Pregnancy (ZIKV-exposed) cohort and the ZIP cohort (ZIKV-unexposed), in Pernambuco, Brazil (2015–2020).

Adverse Outcomes	Total	Case (N/ %)	RR (95% - CI)	P-value	AR%	95% - CI	OR_adjusted*_ (95% - CI)	P-value^a^
Neurologic Abnormalities*								
*Unexposed*	664	14 (**2.11**)	1	–	–	–	–	–
*Positive + Suspected*	252	30 (**11.90**)	5.64 (3.04 – 10.47)	0,000	82%	67% – 90%	6.95ª (3.5 – 13.79)	<0.001
*Positive*	225	24(**10.67**)	5.05 (2.66 – 9.6)	0.000	80%	62% – 89%	6.16ª (3.02 – 12.6)	<0.001
Structural Ophthalmological Abnormalities**								
*Unexposed*	527	19 (**3.61**)	1		–	–	–	–
*Positive + Suspected*	326	4 (**1.23**)	0.34 (0.11 – 0.99)	0.037	–	–	0.17^b^ (0.03 – 0.88)	0.035
*Positive*	291	4 (**1.37**)	0.40 (0.13 – 1.17)	0.083	–	–	0.19^b^ (0.03 – 1.03)	0.055
**Strabismus**								
*Unexposed*	520	4 **(0.77**)	1		–	–	–	–
*Positive + Suspected*	256	2 (**0.78**)	1.01 (0.18 – 5.50)	0.985	15%	-43% - 81%	1.00^b^ (0.17- 5.86)	0.995
*Positive*	229	0	–	–	–	–	–	–
**Nystagmus**								
*Unexposed*	520	1 (**0.19**)	1		–	–	–	–
*Positive + Suspected*	255	2 (**0.78**)	4.10 (0.21 – 242.4)	0.212	75%	-16% - 97%	4.17^b^ (0.35 – 48.7)	0.254
*Positive*	228	0	–	–	–	–	–	–
Failure in the audiological screening test***								
*Unexposed*	560	3 (**0.54**)	1		–	–	–	–
*Positive + Suspected*	223	11 (**4.93**)	9.20 (2.59 – 32.69)	0.000	89%	61% - 96%	8.66 ª (2.34 – 32)	0.001
*Positive*	199	10 (**5.03**)	9.38 (2.60 – 33.73)	0.000	89%	61% -97%	8.68 ª (2.3 – 32.65)	0.001
CNS imaging abnormalities****								
*Unexposed*	387	1 (**0.26**)	1		–	–	–	–
*Positive + Suspected*	228	13 (**5.70**)	22.06 (2.90 – 167.5)	0.000	95%	65% - 99%	12.78^c^ (1.4 – 113.3)	0.022
*Positive*	164	11 (**6.71**)	25.95 (3.37 – 199.4)	0.000	96%	70% - 99%	15.57^c^ (1.7 – 143.4)	0.015

^#^Pearson chi-squared test;

Positive + Suspected - Evidence of infecion: Robuste, moderate, limited evidence + unespecific flavivirus e inconclusive

Positive - Evidence of infecion: Robuste, moderate, limited evidence

*considering irritability, change in tone, dysphagia, or seizure

**considering RetCam findings (fundus of the eye, optic nerve (hypoplasia, pallor or papillary excavation) and retina (pigment dispersion, chorioretinal atrophy, vascular alteration))

***considering BERA

****considering changes in transfontanellar ultrasound (calcification, ventriculomegaly)

^a^adjusted for age, years of education, and recreational drug use

^b^adjusted for years of education, recreational drug use and smoking

^c^adjusted for age, years of education and smoking

Considering ophthalmological alterations, the overall frequency of structural (fundus of the eye, optic nerve and retina) abnormalities was lower in the ZIKV-exposed group than the ZIKV-unexposed group; the RR was 0.34 (95%-CI 0.11 to 0.99), and OR_adj_ was 0.17 (95%-CI 0.03 to 0.88).(Table 3). The analysis of each structural ophthalmological abnormalities separately is shown in S2 Table. All children with optic nerve alterations presented with cupping (3/323 in the ZIKV-exposed group and 16/527 in the ZIKV-unexposed group). No optic nerve hypoplasia was observed in any of the children in either group. Among the three children with retinal alterations in the unexposed group, two cases of chorioretinal atrophy were identifed and one case of pigment dispersion was identified; the one child with retinal alterations in the ZIKV-exposed group presented with chorioretinal atrophy (S2 Table).

A total of 223 children born to ZIKV-exposed mothers underwent the BERA screening and the RR was 9.20 (95% CI 2.59 to 32.69) with an AR% of approximately 90% and the OR_adj_ was 8.66 (95% CI 2.34 to 32) (Table 3).

Relative to the unexposed group, children in the ZIKV-exposed group had 12,78 times (95%-CI 1.4 to 113.3) higher odds of presenting with at least one CNS imaging alteration (i.e., calcification or ventriculomegaly), and the AR% was 95% (Table 3). We observed that approximately 4.4% of children in the ZIKV-exposed group presented with calcifications, whereas in the unexposed group, no child presented with this finding. When calculating the RR for calcifications, a value of 24.5 was obtained (95%-CI 3.92 to ∞). For the occurrence of ventriculomegaly, the RR was 3.39 (95% CI 0.30 to 37.22). Cortical atrophy was not observed in either group.

Table 4 presents the results related to prematurity, weight and viability of the fetus/newborn. No difference was observed in the frequency of these findings in liveborn children born to ZIKV-exposed or unexposed mothers.

**Table 4:** Relative risk, attributable risk percent and adjusted odds ratio and respectives 95% confidence intervals of perinatal outcomes related to Zika virus exposure in pregnancy in the MERG Pregnancy (ZIKV-exposed) cohort and the ZIP cohort (ZIKV-unexposed), in Pernambuco, Brazil (2015–2020).

Perinatal Outcomes	Total (n)	Case (N/ %)	RR (95% - CI)	P-value	AR%	95% - CI	OR_adjusted*_ (95% - CI)	P-valueª
**Low Birth Weight (<2500gr)**								
*Unexposed*	537	42 (**7.82**)	*1*	*–*	–	–	–	–
*Positive + Suspected*	229	21 (**9.17**)	1.17 (0.71 – 1.93)	0.533	14%	-40% - 48%	1.21ª (0.69 – 2.13)	0.496
*Positive*	207	20 (**9.66**)	1.23 (0.74 – 2.05)	0.415	19%	-34% - 51%	1.30ª (0.73 – 2.31)	0.361
**Low Birth Weight (<= 2DP)**								
*Unexposed*	537	13 (**2.42**)	*1*	*–*	–	–	–	–
*Positive + Suspected*	229	9 (**3.9**)	1.62 (0.70 – 3.74)	0.252	38%	-42% - 73%	1.62 ^e^ (0.66 – 3.93)	0.284
*Positive*	207	9 (**4.35**)	1.79 (0.77 – 4.13)	0.164	44%	-28% - 75%	1.82 ^e^ (0.75 – 4.44)	0.183
**Prematurity**								
*Unexposed*	537	43 (**8.01**)	*1*	*–*	–	–	–	–
*Positive + Suspected*	174	17 (**9.77**)	1.22 (0.71 – 2.08)	0.467	18%	-27% - 47%	1.29 ^e^ (0.70 – 2.36)	0.400
*Positive*	155	14 (**9.03**)	1.12 (0.63 – 2.00)	0.682	11%	-57% - 50%	1.15 ^e^ (0.60 – 2.19)	0.669
SGA*								
*Unexposed*	537	53 (**9.87**)	1	–	–	–	–	–
*Positive + Suspected*	229	25 (**10.92**)	1.10 (0.70 – 1.73)	0.660	9%	-41% - 42%	1.03^i^(0.61 – 1.74)	0.887
*Positive*	207	25 (**12.08**)	1.22 (0.78 – 1.91)	0.378	18%	-27% - 47%	1.16^i^ (0.69 – 1.96)	0.556

^#^Pearson chi-square.

Positive + Suspected - Evidence of infecion: Robuste, moderate, limited evidence + unespecific flavivirus e inconclusive.

Positive - Evidence of infecion: Robuste, moderate, limited evidence.

^a^adjusted for age, years of education and recreational drug use.

^b^adjusted for age, years of education and smoking.

^c^adjusted for age, years of education, recreational drug use and smoking.

*small for gestational age.

As demonstrated in Table 5, comparing between children born to ZIKV-exposed and unexposed mothers, the OR_adj_ for presenting with at least one of the abnormalities compatible with CZS (microcephaly, alterations in CNS imaging, neurological alterations, and/or ophthalmological alterations) was 2.46 (95%-CI 1.42 to 4.26) and the AR% was 60%. When we analyzed the presence of concomitant alterations, the RR for the combination of the occurrence of microcephaly, CNS imaging alterations and neurological alterations was 2.45 (95%-CI 0.06 to ∞, p-value 0.577). For the combination of CNS imaging and neurological alterations, the OR was 9.57 (95%-CI 1.02 to ∞). For the combination of neurological alterations and ophthalmological alterations, the RR was 2.45 (95%-CI 0.15 to 39.0) and the AR% was 64%.

**Table 5:** Relative risk, attributable risk percent and adjusted odds ratio, and respective 95% confidence intervals and of having at least one alteration and of concomitant alterations related to exposure to Zika virus during pregnancy in the MERG Pregnancy Cohort and in the Zika in Infants and Pregnancy Cohort, in Pernambuco, Brazil (2015–2020).

Adverse outcomes	Total (n)	Case (N/ %)	RR (95% - CI)	P-value	AR%	95% - CI	OR_adjusted_ (95% - CI)	P-value^#^
Any of the outcomes*								
*Unexposed*	359	36 (**10.03**)	1	–	–	–		
*Positive + Suspected*	146	32 (**21.92**)	2.18 (1.41 – 3.37)	0.000	60%	30% - 77%	2.46ª (1.42 – 4.26)	0.001
*Positive*	128	27 (**21.09**)	2.10 (1.33 – 3.32)	0.001	58%	24% - 76%	2.27ª (1.27 – 4.09)	0.005
**Microcephaly and CNS imaging abnormalities and Neurologic Abnormalities**								
*Unexposed*	359	0	1	–	–	–	–	–
*Positive + Suspected*	146	1 (**0.68**)	2.45^***¥***^ (0.06 - ∞)	0.578	–	–	–	–
*Positive*	128	1 (**0.78**)	2.800^***¥***^ (0.07 - ∞	0.525	–	–	–	–
**CNS imaging abnormalities and Neurologic Abnormalities**								
*Unexposed*	359	0	1	–	–	–	–	–
*Positive + Suspected*	146	3 (**2.05**)	9.57^***¥***^ (1.02 - ∞)	0.047	–	–	–	–
*Positive*	128	3 (**2.34**)	10.93^***¥***^ (1.16 - ∞)	0.035	–	–	–	–
**Neurologic Abnormalities and Ophthalmologic Abnormalities**								
*Unexposed*	359	1 (**0.28**)	1	–	–	–	–	–
*Positive + Suspected*	146	1 (**0.68**)	2.45 (0.15 – 39.0)	0.509	59%	31% - 99%	–	–
*Positive*	128	1 (**0.78**)	2.80 (0.17 – 44.5)	0.445	64%	0.27% -99%	–	–

^#^Pearson chi-square.

Positive + Suspected - Evidence of infecion: Robuste, moderate, limited evidence + unespecific flavivirus e inconclusive.

Positive - Evidence of infecion: Robuste, moderate, limited evidence.

*Microcephaly and/or CNS imaging abnormalities and/or Neurologic Abnormalities and/or Ophthalmologic Abnormalities.

^a^adjusted for age, years of education, recreational drug use and tabagism.

^¥^OR was calculated using a median unbiased estimator for binary data in an unconditional logistic regression model.

A total of 229 women were tested for Rubella, Cytomegalovirus, Parvovirus B19 and Toxoplasmosis in the MERG Pregnancy Cohort, and 295 were tested for Rubella, Cytomegalovirus, and Toxoplasmosis in the ZIP Cohort. The frequency of IgM positivity for these pathogens was very low (i.e., < 1% for all STORCH agents tested), with the exception of Toxoplasmosis in the ZIP Cohort, in which the seroprevalence was 1.4% (S3 Table)

## Discussion

Relative to children without prenatal ZIKV exposure, children born to mothers infected with ZIKV during pregnancy experienced higher risks of microcephaly, neurological, and CNS imaging alterations and failure in the audiological screening test. The magnitude of the association varied across the outcomes, and the adjusted odds ratios were approximately 3-times greater for microcephaly, which had with an AR% of 64%, and 12-times greater for CNS imaging alterations, which had an AR% of approximately 95%.

Published evidence on offspring with RT-PCR confirmed prenatal ZIKV exposure from 13 cohorts in the ZBC-Consortium reported that the absolute risks of microcephaly were 2.6% (95%-CI 1.1 to 4.5) at birth or first assessment and rose to 4.0% (95%-CI 2.0 to 6.6) when any time during follow-up was considered [5]. Further, the absolute risk of neurological alterations was 18.7% (95%-CI 2.6 to 41.5) and was 7.9% (95%-CI 2.8 to 14.7) for CNS imaging alterations. When considering at least one alteration, the overall risk was 24.7% (95%-CI 0.10 to 63.6) [5,6].

In this study, microcephaly was the most serious clinical manifestation observed, but not the most common. Different studies have reported disparate results regarding the magnitude of the association between prenatal ZIKV exposure and microcephaly. This may reflect, in part, differences in the timing of ZIKV infection during pregnancy [4,15–17]. The results found in our study corroborate the findings of Brasil, et al., and Sanchez Clemente, et al. [4,5], who observed a higher risk of microcephaly in children born to mothers exposed to ZIKV during pregnancy; however, in their studies the differences between the ZIKV-positive and ZIKV-negative groups were not statistically significant, which may be due to the relatively small sample size of the individual studies. By contrast, Funk, et al., (2016) reported a higher frequency of microcephaly in children born to ZIKV-unexposed mothers; however, this finding is possibly due to methodological issues, mainly the fact that pregnant women in the unexposed group were only tested for ZIKV using serology at the time of birth [6]. Some authors have indicated that even for women with a positive RT-PCR result, there is a significant proportion who do not seroconvert [10]; therefore, as the study by Funk and colleagues relied on immunological evidence it is possible that there was a proportion of ZIKV-exposed women who were misclassified as being unexposed [6b]. It is of note that our study corroborates the findings of a case-control study conducted by Araújo et al (2016) [1]. In this study, newborns with microcephaly (cases) were compared to newborns without microcephaly (controls) in relation to evidence of exposure to ZIKV infection during pregnancy, and it was found that the odds of microcephaly were at least 13-times greater in the ZIKV-infected children [1]. The study of Sanchez Clemente et al. (2020) also found evidence of an association between microcephaly and prenatal ZIKV infection in children infants [5]. Another strength of our study is that the AR% was calculated. The AR% expresses the percentage of the cases among exposed group that can be attributed to the exposure, meaning, in our results, that approximately 70% of cases of microcephaly among children born to mothers exposed to ZIKV may be attributed to the ZIKV exposure.

Among the ten ZIKV-unexposed children with microcephaly using the -2SD cut-off, five were considered borderline, with head circumferences ranging from -2.01 to -2.35SD, which may reflect inaccuracies in the measurement of head circumference. Furthermore, as the definitions for microcephaly are based on Z-scores for head circumferences from growth curves, we expect a fraction of children will have smaller head circumferences as part of the normal distribution. An alternative explanation for the occurrence of microcephaly in the ZIKV-unexposed group would be the occurrence of another congenital infection. Five of the ten children who were diagnosed with microcephaly in the ZIKV-unexposed group were born to mothers who tested IgG-positive for Rubella, Cytomegalovirus, Toxoplasmosis and/or Herpes; notably, none tested IgM-positive for any of these infections, so we cannot discern whether the infections occurred during or prior to pregnancy. The other five children and their mothers were not tested for STORCH pathogens, but one had a genetic disorder related to chromosome 10, and another was diagnosed with Edwards Syndrome (trisomy 18), which includes microcephaly in the clinical presentation.

In our study, the OR_adj_ of neurological abnormalities, including irritability, seizures, tonus alterations and dysphagia, was 6.95, comparing ZIKV-exposed and unexposed children. We discovered no estimates in the published literature of the risk of these alterations relative to ZIKV exposure status. Our findings suggest that 82% of neurological alterations in children born to positive mothers could be attributed to the prenatal ZIKV infection.

Ocular abnormalities are identified in a large proportion (31.6%) of the ZIKV-exposed children with microcephaly [19]. However, contrary to expectations, the odds of ophthalmological abnormalities were not elevated when comparing between children born to mothers exposed and unexposed to ZIKV infection [18]. The frequency of ophthalmological abnormalities among children of ZIKV-exposed mothers in this study differs from the findings of a previous article on the MERG Cohort published by our group [19]. This may have been due to the fact that, in an attempt to harmonize with the ZIP Cohort, we defined ophthalmological abnormality based on a different assessment technique. In the previous study, we considered the presence of an abnormality as detected by RetCam or fundoscopy, which may have increased the possibility of identifying alterations, whereas the current study only used the results obtained from RetCam [11,19]. The high frequency of increased optic nerve cupping in children born to unexposed mothers is also notable, and we considered alternative risk factors, such as STORCH agents, systemic diseases and prematurity. With regard to STORCH agents, we discovered no reports in the literature of an association between congenital infection and increased optic nerve cupping. Systemic diseases (Solomon’s syndrome (epidermal nevus syndrome), Wolf-Hirschhorn syndrome, etc.) that may lead to this manifestation are rare [20] and have not been diagnosed in these children born to ZIKV-unexposed mothers. Prematurity also does not seem to be an explanation for this finding, since only one of these children was premature. Another possibility is that these cuppings are just a physiological variation (physiological cupping) [18]. Nystagmus was more frequent among children born to ZIKV-exposed mothers, although with no statistical significance.

We observed a greater frequency of ‘failing’ in the auditory brainstem response test (BERA) in children born to ZIKV-exposed mothers; however, prior studies are difficult to compare, since these findings have been primarily described in children with microcephaly. Furthermore, these results need to be interpreted with caution since they were based on a screening test and most children (9 out of 11 children born to ZIKV-exposed mothers) did not fail when retested. Factors, such as excessive environmental noise, sleep state or alertness of the newborn, and external auditory canal obstruction by vernix or wax and effusion in the middle ear, can contribute to the high rate of retests in neonatal hearing screening programs [21]. The ‘failure’ rate in screening programs should not exceed 10% [22].

The adjusted odds ratio of CNS imaging abnormalities were nearly 13-times greater in the ZIKV-exposed children than the unexposed children, and calcifications were detected exclusively in the exposed group. Similarly, Brasil, et al., (2016) described CNS imaging abnormalities (i.e., cerebral calcifications, increased ventricular dilation and hypoplasia in several brain structures) only in children born to mothers exposed to ZIKV [4]. The AR% for CNS imaging alterations in our study was 95% (95%-CI 65–99), surpassing the AR% for the occurrence of microcephaly, which was 72% (95%-CI 41–86).

When we assessed the set of congenital manifestations potentially associated with ZIKV infection during pregnancy, we observed that the risk of presenting with at least one manifestation was approximately 2-times greater in children born to exposed mothers. In this study, very few children in the ZIKV-exposed group presented with manifestations that occurred concomitantly. The risk of having concurrent abnormalities was also shown to be low in the ZBC-Consortium IPD-MA; only 3.7% of the children with RT-PCR confirmed prenatal Zika virus exposure had both neuroimaging and neurological abnormalities and only 1.9% had both neurological and ophthalmic abnormalities [8].

Overall, we observed no association between prenatal ZIKV exposure and adverse birth outcomes (low birth weight, prematurity and SGA). Although the occurrence of SGA children has been reported by other studies as a possible outcome of prenatal ZIKV infection [4], this association was not observed in our study.

## Advantages and limitations

This study presents advantages and limitations. One of the limitations is that the comparison group is external (i.e., a cohort of ZIKV-exposed mothers (MERG Pregnancy Cohort) was compared with another cohort of mothers unexposed to ZIKV (ZIP Cohort). Notably, we observed differences in some characteristics between the exposed and unexposed pregnant women. To account for these differences, we adjusted the association for these factors. However, we cannot exclude that there may have been some residual confounding. It should be noted that the cohort of unexposed mothers was followed in the same location (Recife and neighboring cities) and that the pregnant women and children were assessed by the same research group (MERG). The periods of data collection for both cohorts only partially overlapped, but the time window in which the data was collected (2015–2018) is not large, and therefore it would not be expected important changes in the frequency of other potential confounders or on the socioeconomic conditions.

A strength of this study is that pregnant women in the ZIP Cohort were recruited in the first half of pregnancy (i.e., up to the 17th week of gestation) and were systematically tested for anti-ZIKV IgM Antibodies or viral RNA (by RT-PCR) throughout their pregnancy. This intensive testing increased the specificity for identifying truly negative controls in the unexposed group and provided the best comparison group among those referred to in the literature. One limitation is the potential occurrence of a classification error regarding the presence of microcephaly. As the fieldworkers were not aware of the exposure status of the mothers, it is likely that misclassification, if it occurred, was non-differential, which may have led to underestimation of the association. To reduce the likelihood that children without microcephaly will be mistakenly diagnosed as having this condition, we performed a sensitivity analysis using -2.5SD and -3SD as alternative cutoff points. The results obtained have reinforced the existence of an association between microcephaly and prenatal exposure to ZKV.

Another limitation is that there was a difference in the assessment age of children born to exposed and unexposed mothers. To minimize this problem, we analyzed data either at birth or at the first assessment for children in the MERG cohort, and for children in the ZIP cohort, we analyzed data either at birth or at the assessment at an age closest to that at which the assessment was carried out with the MERG children; in addition, we adjusted for children’s age in the analysis. Another limitation concerns the STORCH agents, since not all mothers in either the exposed or unexposed groups had their samples tested for these pathogens. Of note, testing for STORCH agents was not based on clinical indication, but instead reflected operational reasons. Therefore, there is no reason to think that selection bias was introduced in the selection of those that who were tested, so we assume the tested subsample is representative of participants in both cohorts. It is worth noting that the frequency of IgM positivity was very low for all STORCH tested and the of IgM for Rubella, Cytomegalovirus and Toxoplasmosis were similar in both cohorts and was not associated with socioeconomic status.

## Conclusion

Our results provide new insights into the relative and attributable risks related to ZIKV infection during pregnancy. We conclude that the risk of congenital abnormalities is greater among children born to mothers exposed to ZIKV than in children born to mothers who were not exposed, and that a high proportion of abnormalities found in children born to exposed mother can be attributed to ZIKV exposure. Our findings provide robust evidence that, relative to unexposed children, children with prenatal exposure to ZIKV have a greater risk of microcephaly, neurological alterations, failure in the audiological screening test, and CNS imaging alterations. We found that at least 39% of cases of microcephaly, 68% of cases with neurological alterations, and 70% of cases with CNS imaging alterations among children born to exposed mothers may be attributed to the prenatal ZIKV infection. In addition to a higher risk of microcephaly, children exposed to ZIKV in utero also had a higher risk of other abnormalities. Overall, these findings underscore the importance of continuing the follow-up of these children to evaluate the long-term consequences of ZIKV infection during pregnancy and reinforce the need to rapidly develop a safe and effective vaccine to prevent congenital ZIKV infections.

## Supporting information

S1 Table Relative risk and attributable risk percent and respective 95% confidence intervals for neurologic abnormalities related to Zika virus exposure during pregnancy in the MERG Pregnancy Cohort (ZIKV-exposed) and the ZIP Cohort (ZIKV-unexposed), in Pernambuco, Brazil (2015–2020). (DOCX)

S2 Table Relative risk, attributable risk percent and respective 95% confidence intervals for ophthalmological abnormalities related to Zika virus exposure during pregnancy in the MERG Pregnancy Cohort (ZIKV-exposed) and the ZIP Cohort (ZIKV-unexposed), in Pernambuco, Brazil (2015–2020). (DOCX)

S3 Table STORCH infections during pregnancy in the MERG Pregnancy (ZIKV-exposed) cohort* and in the ZIP cohort (ZIKV-unexposed). (DOCX)
