# Supplementary material for: Relative and attributable risks of neurological and perinatal adverse outcomes among children with and without prenatal Zika virus exposure in Northeast Brazil: A prospective cohort study (2015–2018)
Source: PLoS Negl Trop Dis. 2025 Aug 8;19(8):e0013344. doi: 10.1371/journal.pntd.0013344 (PMC12334026; doi:10.1371/journal.pntd.0013344)
Supplement: S1 Table — (DOCX) [file pntd.0013344.s001.docx]

**S1 Table. Relative risk and attributable risk percent and respective 95% confidence intervals for neurologic abnormalities related to Zika virus exposure during pregnancy in the MERG Pregnancy Cohort (ZIKV-exposed) and the ZIP Cohort (ZIKV-unexposed), in Pernambuco, Brazil (2015–2020).**

| Neurologic Abnormalities | Total | Case  (n / %) | RR  (95% - CI) | P-value | AR% | | 95% - CI |
| --- | --- | --- | --- | --- | --- | --- | --- |
| **Dysphagia** |  |  |  |  |  |  |  |
| Unexposed | 666 | 5 (**0.75**) | 1 | - | | - | - |
| Positive + Suspected | 254 | 17 (**6.69**) | 8.91  (3.32 – 23.91) | <0.000 | | 88% | 69% -95% |
| Positive | 227 | 14 (**6.14**) | 8.21  (2.99 – 22.55) | <0.000 | | 87% | 66% - 96% |
| **Irritability** |  |  |  |  |  |  |  |
| Unexposed | 665 | 4 (**0.60**) | 1 |  | |  |  |
| Positive + Suspected | 253 | 9 (**3.56**) | 5.91  (1.83 – 19.03) | <0.000 | | 83% | 45% - 94% |
| Positive | 226 | 8 (**3.54**) | 5.88  (1.78 – 19.35) | <0.000 | | 83% | 44% - 94% |
| **Seizure** |  |  |  |  |  |  |  |
| Unexposed | 665 | 3 (**0.45**) | 1 | - | | - | - |
| Positive + Suspected | 258 | 3 (**1.16**) | 2.57  (0.52 – 12.68) | 0.227 | | 61% | 9,0% - 92% |
| Positive | 229 | 2 (**0.87**) | 1.91  (0.32 – 11.41) | 0.466 | | 47% | 2.09% - 91.2%) |
| **Tonus alterations** |  |  |  |  |  |  |  |
| Unexposed | 666 | 3 (**0.45**) | 1 | - | | - |  |
| Positive + Suspected | 258 | 9 (**3.49**) | 7.98  (1.96 – 46.11) | <0.000 | | 87% | 49% - 97% |
| Positive | 231 | 6 (**2.60**) | 5.89  (1.24 – 36.62) | 0.004 | | 83% | 19% - 97% |

Positive + Suspected - Evidence of infecion: Robuste, moderate, limited evidence + unespecific flavivirus e inconclusive

Positive - Evidence of infecion: Robuste, moderate, limited evidence
