# Supplementary material for: Relative and attributable risks of neurological and perinatal adverse outcomes among children with and without prenatal Zika virus exposure in Northeast Brazil: A prospective cohort study (2015–2018)
Source: PLoS Negl Trop Dis. 2025 Aug 8;19(8):e0013344. doi: 10.1371/journal.pntd.0013344 (PMC12334026; doi:10.1371/journal.pntd.0013344)
Supplement: S2 Table — (DOCX) [file pntd.0013344.s002.docx]

**S2 Table. Relative risk, attributable risk percent and respective 95% confidence intervals for ophthalmological abnormalities related to Zika virus exposure during pregnancy in the MERG Pregnancy Cohort (ZIKV-exposed) and the ZIP Cohort (ZIKV-unexposed), in Pernambuco, Brazil (2015–2020)**

| Ophthalmological Abnormalities | Total | Case  (n / %) | RR  (95% - CI) | P-value |  |
| --- | --- | --- | --- | --- | --- |
| **Fundus of the eye** |  |  |  |  |  |
| Unexposed | 526 | 31 (5.89) | 1.0 | - | |
| Positive + Suspected | 261 | 3 (1.15) | 0.19  (0.06 – 0.63) | 0.002 | |
| Positive | 234 | 3 (1.28) | 0.21  (0.06 – 0.70) | 0.004 | |
| **Optive nerve –** hypoplasia*, pallor, cupping** |  |  |  |  |  |
| Unexposed | 527 | 16 (**3.04**) | 1.0 | - | |
| Positive + Suspected | 326 | 3 **(0.92**) | 0.30  (0.09 – 1.03) | 0.042 | |
| Positive | 291 | 3 **(0.92**) | 0.34  (0.10 – 1.15) | 0.068 | |
| **Optive nerve** hypoplasia*, pallor |  |  |  |  |  |
| Unexposed | 527 | 1 (**0.19**) | 1.0 | - | |
| Positive + Suspected | 326 | 0 (0) | - | - | |
| Positive | 291 | 0 (0) | - | - | |
| **Retinal Abnormalities** |  |  |  |  |  |
| Unexposed | 527 | 3***^¥^*** (**0.57**) | 1.0 | - | |
| Positive + Suspected | 326 | 1^#^ (**0.31**) | 0.54  (0.06 – 5.16) | 0.585 | |
| Positive | 291 | 1 (**0.34**) | 0.60  (0.06 – 5.78) | 0.658 | |
| **Optive nerve^╦^ and retinal abnormalities** |  |  |  |  |  |
| Unexposed | 527 | 4 (**0.76**) | 1.0 | - | |
| Positive + Suspected | 326 | 1 (**0.31**) | 0.40  (0.05 – 3.60) | 0.400 | |
| Positive | 291 | 1 (**0.34**) | 0.45  (0.05 – 4.03) | 0.466 | |

Positive + Suspected - Evidence of infecion: Robuste, moderate, limited evidence + unespecific flavivirus e inconclusive

Positive - Evidence of infecion: Robuste, moderate, limited evidence

*No optic nerve hypoplasia was observed

** children with optic nerve alterations presented with cupping

¥ Two cases of chorioretinal atrophy and one case of pigment dispersion

# Chorioretinal atrophy

^╦^ Hypoplasia, pallor
