# Supplementary material for: Relative and attributable risks of neurological and perinatal adverse outcomes among children with and without prenatal Zika virus exposure in Northeast Brazil: A prospective cohort study (2015–2018)
Source: PLoS Negl Trop Dis. 2025 Aug 8;19(8):e0013344. doi: 10.1371/journal.pntd.0013344 (PMC12334026; doi:10.1371/journal.pntd.0013344)
Supplement: S3 Table — (DOCX) [file pntd.0013344.s003.docx]

**S3 Table. STORCH infections during pregnancy in the MERG Pregnancy (ZIKV-exposed) cohort* and in the ZIP cohort (ZIKV-unexposed)**

| **Infection** | **Positive + Flavivirus + inconclusive** | **ZIKV-unexposed** | **p-valor** |
| --- | --- | --- | --- |
|  |  |  |  |
| STORCH |  | | |
| **Rubella** |  |  |  |
| IgM | 0/227 (0) | 1/292 (0.3) | 0.527 |
| IgG | 195/220 (88.6) | 278/295 (94.2) | 0.024 |
| **Cytomegalovirus** |  | | |
| IgM | 1/229 (0.4) | 1/294 (0.3) | 0.876 |
| IgG | 215/227 (94.7) | 288/295 (97.6) | 0.078 |
| DNA | 4/56 (7.1) | - | - |
| **Parvovirus** |  | | |
| IgM | 1/223 (0.4) | - | - |
| IgG | 160/217 (73.7) | - | - |
| **Toxoplasmosis** |  | | |
| IgM | 1/205 (0.5) | 4/291 (1.4) | 0.382 |
| IgG | 141/205 (68.8) | 169/295 (57.3) | 0.009 |
| **Herpes 1** |  |  |  |
| IgM | - | - | - |
| IgG | - | 241/294 (82.0) | - |
| **Herpes 2** |  |  |  |
| IgM | - | - | - |
| IgG | - | 103/292 (35.3) | - |

*Adapted from Ximenes RAdA, Miranda-Filho DdB, Montarroyos UR, Martelli CMT, Araújo TVBd, Brickley E, et al. (2021) Zika-related adverse outcomes in a cohort of pregnant women with rash in Pernambuco, Brazil. PLoS Negl Trop Dis 15(3): e0009216. <https://doi.org/10.1371/journal>. pntd.00092167
